# The pattern of medication use, and determinants of the prevalence of polypharmacy among patients with a recent history of depressive disorder: results from the pars cohort study

**DOI:** 10.1186/s40359-022-00716-9

**Published:** 2022-01-18

**Authors:** Mehrnoosh Ghaed-Sharaf, Sanam Hariri, Hossein Poustchi, Maryam Nourollahi, Sara Khani, Erfan Taherifard, Zahra Mohammadi, Maryam Hadipour, Rasoul Sabaei, Abdullah Gandomkar, Fatemeh Malekzadeh, Hossein Molavi Vardanjani

**Affiliations:** 1 MPH Department, School of Medicine, Shiraz University of Medical Sciences, Shiraz, Iran; 2 Liver and Pancreatobiliary Diseases Research Center, Digestive Diseases Research Institute, Shariati Hospital, Tehran University of Medical Sciences, Tehran, Iran; 3 Liver and Pancreatobiliary Diseases Research Center, Digestive Diseases Research Institute, Tehran University of Medical Sciences, Tehran, Iran; 4 Student Research Committee, Shiraz University of Medical Sciences, Shiraz, Iran; 5 Health Policy Research Center, Institute of Health, Shiraz University of Medical Sciences, Shiraz, Iran; 6 Department of Psychology, Faculty of Medicine, Najafabad Branch, Islamic Azad University, Isfahan, Iran; 7 Non-Communicable Disease Research Center, Shiraz University of Medical Sciences, Shiraz, Iran; 8 Digestive Diseases Research Center, Digestive Diseases Research Institute, Shariati Hospital, Tehran University of Medical Sciences, Tehran, Iran; 9 MPH Department, School of Medicine, Research Center for Traditional Medicine and History of Medicine, Shiraz University of Medical Sciences, Shiraz, Iran

**Keywords:** Polypharmacy, Depressive disorders, Medication use, Antidepressants, Prevalence

## Abstract

**Background:**

Inappropriate medication use among individuals with depressive disorders (DD) is a rising public health challenge. We aimed to investigate the polypharmacy and its determinants among individuals with DD in a less developed region, and evaluate the pattern of medication use in this population.

**Methods:**

Data was extracted from Pars Cohort Study (PCS) between 2016 and 2019. Participants were asked to bring all the medication they were using regularly, and history of DD during the last 12 months prior to study was obtained. The Anatomical Therapeutic Chemical classification was applied and polypharmacy was defined as concurrent use of five or more medications. Logistic regression models were developed to estimate the associations between polypharmacy and DD, adjusted for relevant covariates. The prevalence of consumption of each drug class was estimated among males, females, and elders. Logistic regression was applied and the adjusted odds ratio (OR) and its 95% confidence interval (CI) were estimated.

**Results:**

A total of 9264 participants with a mean age of 52.6 (SD: 9.7) were enrolled. The prevalence of polypharmacy was 22.6% [95% CI (20.7–24.6)]. The most common drug classes were genitourinary system (55.4%) and nervous system (29.1%) medication, respectively. Recent history of DD was reported among 19.4% (n = 1795) participants, the majority of whom were females. Factors associated with polypharmacy include female gender (OR: 1.51), Fars ethnicity (OR: 1.52), lower physical activity (OR: 1.74), and higher socioeconomic status (OR: 1.40). The prevalence of antidepressant use among males was higher than females (*P* < 0.001).

**Conclusion:**

The prevalence of polypharmacy is high among patients with a recent history of depressive disorder. Females, individuals with higher socioeconomic status and lower physical activity, and those who use tobacco are more likely to be polymedicated. Surveillance measures need to be established to monitor the patterns of medication use among individuals with depressive disorders.

## Introduction

Polypharmacy is a growing public health global challenge [1]. Although polypharmacy may be necessary and sometimes inevitable, it may be an evidence of inappropriate medication use in a considerable proportion of patients suffering from it [1, 2]. There is a huge body of evidence on the prevalence of polypharmacy, and its correlates and consequences [3, 4]. However, the most of studies are from developed regions and are focused on the elderly populations, therefore our knowledge about polypharmacy may not be generalizable to the younger populations who are from developing or less developed regions [5, 6].

Polypharmacy is a multifactorial iatrogenic condition but not a single-cause phenomenon. It may be a result of healthcare system-related, contextual factors and patient-related factors. The contextual factors consist of inappropriate interaction between the health professionals, absence of effective strategies to prevent inappropriate polypharmacy, allowed direct-to-consumer marketing, the medicalization of everyday life, publicly funded healthcare, and culture of single-condition guideline-based prescribing. The patient-related factors include healthcare-seeking behaviors, health literacy, socioeconomic status, self-medication behavior, educational level, gender, age, and number and type of comorbidities [1, 6–8].

Depressive disorders (DD) are highly correlated with the prevalence of polypharmacy and inappropriate medication use [7, 8]. Some authors argued that the correlation of DD and polypharmacy may be bidirectional [9]. DD may result in multimorbidity which is a major driver of polypharmacy, and on the other hand, the polypharmacy may result in the DD [6, 8, 10]. Although a study has shown that the correlation of DD and the polypharmacy is fully mediated by the multimorbidity [11], some other studies showed that DD may be still correlated with the increased prevalence of the polypharmacy even after adjustment for the number and type of comorbidities [6, 8, 10]. These studies have reported a 2–10 times higher prevalence of polypharmacy in people with DD compared to those without DD, after adjustment for multimorbidity, and accordingly, people with DD from different settings are differently at-risk of polypharmacy.

Polypharmacy may result in a higher rate of non-adherence to medication therapy and consequently lead to a treatment failure and higher DD relapse rates [12–14]. Moreover, the polypharmacy complicates the patient’s treatment [1, 7, 12], and leads to adverse conditions such as drug-drug reaction, medication errors, adverse drug side-effects, therapeutic duplication, and unnecessary hospitalizations especially when it is missed by physiatrists while prescribing antidepressants [15]. Therefore, identification of patients with a higher risk of polypharmacy among those with DD could be a fundamental step to improve the quality of healthcare for patients with DD.

Inspired by the fact that the polypharmacy among non-elderly people with depression is not routed in a similar list of risk factors associated with the polypharmacy in people without the DD, or in elderly individuals with or without the DD, we aimed to investigate the correlations of the polypharmacy in a population-based sample of patients with a recent history of the DD. We also aimed to describe the pattern of medication use and prevalence of use of antidepressants among them. We analyzed the baseline data of a large-scale cohort study in a less developed region in southwestern Iran.

## Methods

### Study setting

Baseline data was extracted from the Pars Cohort Study (PCS), which is a population-based 10-year cohort launched from 2016 in southwestern Iran. The study site was a semi-urban area in Fars province named Baladeh (Kazeroun County), and almost all adult inhabitants of this area were invited to participate. Baladeh is a multi-ethnic region that is home to around 40,000 inhabitants; 10,000 of whom are older than 40 years old [16]. Although the public health sector, including primary healthcare centers, is the main sector responsible for the healthcare provision, some self-employed general physicians are also working in this region. However, no specialist or subspecialist healthcare services are available, and inhabitants need to seek such services in the nearest neighboring city (Kazeroun) or the capital city of Fars province (Shiraz).

Inhabitants who were interested to participate in this study were asked to come to the PCS center. Data collection included a comprehensive face-to-face interview and physical examination, anthropometric measurements, and biological sampling, which was done by well-trained local physicians and nurses. Data collection was done using calibrated equipment, and the standard questionnaire that had been developed and used in the PERSIAN Cohort study [17]. More information on the PCS design and procedures have been detailed elsewhere [16].

### Data collection

Participants were asked “Has your physician told you that you have a depressive disorder and you need treatment for that during the last 12 months?”, and if they answered “Yes”, they were classified as having a recent history of depression.

Participants were asked to bring all the medication that they were using regularly at the time of the interview. A trained nurse was responsible to check if each of the drugs is being used by the participant for at least 3 months prior to the interview. Data on the currently used drugs, including over the counters (OTC) and complementary products, was recorded and polypharmacy was defined as using five or more different medications concurrently.

### Drug classification

The Anatomical Therapeutic Chemical (ATC) classification system [18] was used to classify all drugs, except for complementary medicines. The first level of the ATC classification system was used for all drugs. We used the ATC classification system to classify antidepressants (ATC code: N06) which is a subgroup of the nervous system drug class.

### Polypharmacy determinants

Variables included in the analyses were age, gender, marital status, ethnicity, educational level, socioeconomic status (SES), physical activity, body mass index (BMI), cigarette smoking, tobacco smoking, alcohol consumption, multi-morbidity (number of chronic morbidities), metabolically healthy overweight, and metabolically healthy obesity.

Participants without metabolic syndrome (MetS) who were overweight were classified as healthy overweight, and obese participants without MetS were classified as healthy obese. MetS was defined based on the criteria recommended by Alberti et. al. for Asians [19]. SES was determined based on a latent variable measured by analysis of the participants' assets using Multiple Correspondence Analysis (MCA), and participants were categorized into quartiles of this variable (low, low-middle, middle-high, and high). Physical activity was measured by estimating the metabolic equivalent of task (METs), using data collected based on the International Physical Activity Questionnaire (IPAQ), and participants were ranked and categorized into three tertiles of METs (low, moderate, high).

Data on marital status (single, divorced, widowed, married); educational level (illiterate, literate); and ethnicity (Fars, non-Fars) were collected. BMI was calculated and categorized based on the WHO recommendations [17]. Consecutive consumption of tobacco and cigarette smoking for at least six month throughout the participants’ life was defined as ever use. Current use was defined as a weekly consumption in the last six months was defined as current use. Measurement details are available in the PCS cohort profile [16].

Multi-morbidity was addressed based on the number of chronic conditions that a participant currently encounters, and the presence of these chronic disorders is mostly determined by the participant self-reporting of conditions that were already diagnosed by their physicians. These conditions include diabetes mellitus, stroke, cancer, chronic lung disease, atherosclerotic cardiovascular disease, hypertension, chronic liver disease, end-stage renal failure, major depressive disorder, and general anxiety disorder. We also included severe gastroesophageal reflux disease (GERD), Irritable bowel syndrome (IBS), and functional constipation based on clinical criteria that are presented elsewhere [20]. Multi-morbidity was defined as having two or more chronic morbidities.

### Statistical analysis

Mean ± standard deviation (SD) and frequency were used to describe the data were appropriate. The prevalence of polypharmacy and its 95% confidence interval (CI) was estimated assuming the Poisson distribution. The age-standardized prevalence rate (ASR) of polypharmacy was estimated based on the world standard population (WHO 2000–2025). Univariate analyses were performed using the chi-square test. Multivariable binary logistic regression was applied to investigate the association between different independent variables and the prevalence of polypharmacy. The variable selection for multivariable modeling was done based on a univariate P-value of less than 0.3. The final multivariable model was fitted applying a backward elimination technique. Adjusted odds ratios (OR) and their 95% CI were estimated. A two-sided P-value of less than 0.05 was defined as a statistically significant level. Data analysis was done using Stata software (Release 11, College Station, TX: Stata Corp LLC).

## Results

A total of 9264 participants were included in the study; the mean age was 52.6 ± 9.7 years and 54% were female. Overall, 1795 (19.4%) individuals with a mean age of 53.3 ± 9.5 years reported having a depressive disorder during the last 12 months, the majority of whom were females (70.0%).

The overall prevalence of polypharmacy among participants with and without a recent history of DD was 22.6% (95% CI 20.7%, 24.6%) and 7.5% (95% CI 6.9%, 8.1%), respectively (*P* < 0.001). Age-standardized prevalence of polypharmacy among participants with and without a recent history of DD was 23.45 (95% CI 21.0%, 25.7%) and 8.2% (95% CI 7.4%, 8.9%), respectively. Accordingly, the prevalence of polypharmacy in participants with a recent history of DD was significantly higher than those without such history (OR: 3.62, 95% CI 3.15, 4.16). Age and gender-adjusted OR for comparing the prevalence of polypharmacy among participants with and without a recent history of depression was estimated at 22.7 (95% CI 16.9, 30.5).

The prevalence of polypharmacy was significantly higher among participants with a recent history of DD compared to those without such history across different subpopulations. This prevalence was 13.2% (95% CI 10.3%, 16.0%) among male participants with DD and 3.6% (95% CI 3.0%, 4.1%) among males without DD (Table 1).

**Table 1:** Characteristics of PCS participants and prevalence of polypharmacy, categorized by recent history of depressive disorder

Variable	With a recent history of depression		Without a recent history of depression		*P* value
	Number (%)	Prevalence of polypharmacy (95% CI)	Number (%)	Prevalence of polypharmacy (95% CI)	
Gender					
Male	539 (30.0)	13.2 (10.3, 16.0)	3736 (50.0)	3.6 (3.0, 4.1)	< 0.001
Female	1256 (70.0)	26.7 (24.2, 29.1)	3731 (50.0)	11.4 (10.4, 12.4)	< 0.001
Age (years)					
< 50	745 (41.5)	17.5 (14.7, 20.2)	3470 (46.5)	5.2 (4.5, 6.0)	< 0.001
50–59	555 (31.0)	22.2 (18.7, 25.6)	2253 (30.1)	8.0 (6.9, 9.2)	< 0.001
≥ 60	495 (27.5)	31.0 (26.8, 35.0)	1744 (23.4)	11.1 (9.7, 12.7)	< 0.001
Education					
Illiterate	1004 (55.9)	25.4 (22.7, 28.1)	3534 (47.3)	8.7 (7.8, 9.7)	< 0.001
Literate	791 (44.1)	19.1 (16.3, 21.8)	3933 (52.7)	6.3 (5.6, 7.1)	< 0.001
Ethnicity					
Fars	1044 (58.2)	25.5 (22.8, 28.1)	4171 (55.9)	8.6 (7.8, 9.5)	< 0.001
Non-Fars	751 (41.8)	18.6 (15.8, 21.4)	3296 (44.1)	6.0 (5.2, 6.8)	< 0.001
Marital status					
Married	1558 (86.6)	21.6 (19.5, 23.6)	6949 (93.1)	7.2 (6.6, 7.8)	< 0.001
Divorced/widowed	237 (13.2)	29.5 (23.7, 35.4)	515 (6.9)	11.3 (8.5, 14.0)	< 0.001
Socioeconomic status					
Low	501 (28.1)	20.5 (17.0, 24.1)	1907 (25.5)	5.4 (4.4, 6.4)	< 0.001
Low-middle	486 (27.2)	24.5 (20.6, 28.3)	2004 (27.0)	7.3 (6.1, 8.4)	< 0.001
Middle-high	398 (22.3)	20.6 (16.6, 24.6)	1645 (22.1)	8.6 (7.3, 9.9)	< 0.001
High	400 (22.4)	25.0 (20.7, 29.3)	1891 (25.4)	8.8 (7.5, 10.0)	< 0.001
Physical activity					
Low	674 (37.5)	31.0 (27.5, 34.5)	2386 (32.0)	11.0 (9.7, 12.2)	< 0.001
Moderate	619 (34.5)	20.5 (17.3, 23.7)	2437 (32.6)	8.0 (7.0, 9.0)	< 0.001
High	502 (28.0)	13.9 (10.9, 17.0)	2644 (35.4)	3.8 (3.1, 4.5)	< 0.001
Body mass index (BMI) (kg/m2)					
BMI < 25	784 (44.0)	16.4 (13.8, 19.0)	3318 (44.6)	4.8 (4.1, 5.6)	< 0.001
25 < BMI < 3 0	660 (37.0)	25.6 (22.3, 28.9)	2780 (37.4)	7.8 (6.8, 8.8)	< 0.001
BMI > 30	337 (19.0)	31.1 (26.2, 36.1)	1338 (18.0)	13.4 (11.5, 15.2)	< 0.001
Tobacco use					
No	994 (55.4)	18.8 (16.4, 21.2)	4715 (63.3)	6.4 (5.7, 7.1)	< 0.001
Ever use	799 (44.6)	27.3 (24.2, 30.4)	2738 (36.7)	9.3 (8.2, 10.4)	< 0.001
Alcohol use					
No	1752 (97.6)	22.8 (20.9, 24.8)	7314 (97.9)	7.6 (7.0, 8.2)	< 0.001
Ever use	43 (2.4)	13.9 (3.2, 24.8)	153 (2.1)	2.0 (0.0, 4.2)	0.001
Cigarette smoking					
No	1489 (82.9)	24.9 (22.6, 27.0)	5856 (78.4)	8.4 (7.7, 9.2)	< 0.001
Ever smoking	306 (17.1)	11.8 (8.1, 15.4)	1611 (21.6)	3.9 (3.0, 4.8)	< 0.001
Healthy overweight					
No	1502 (83.7)	24.4 (22.2, 26.6)	5943 (79.6)	8.4 (7.7, 9.1)	< 0.001
Yes	293 (16.3)	13.3 (9.4, 17.2)	1524 (20.4)	3.9 (2.9, 4.8)	< 0.001
Healthy obesity					
No	1677 (93.4)	22.7 (20.7, 24.7)	6893 (92.3)	7.6 (7.0, 8.2)	< 0.001
Yes	118 (6.6)	21.2 (13.7, 28.7)	574 (7.7)	5.7 (3.8, 7.6)	< 0.001
Multi-morbidity					
No	63 (3.5)	100.0 (-)	3960 (53.0)	2.1 (1.6, 2.5)	< 0.001
Yes	1732 (96.5)	23.4 (21.4, 25.4)	3507 (47.0)	13.6 (12.4, 14.7)	< 0.001

CI: confidence interval

**Fig. 1:**
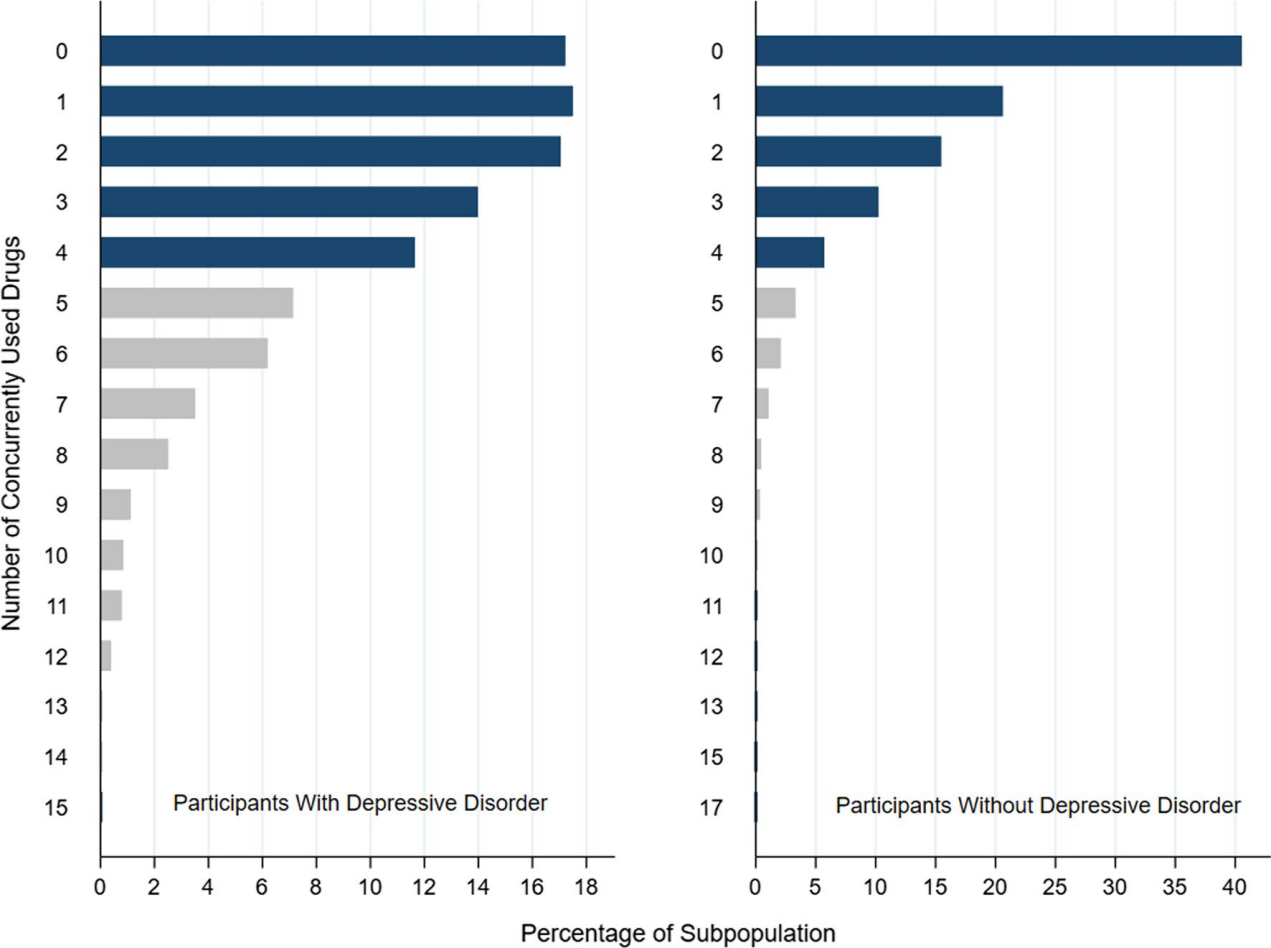
Number of concurrently used drugs categorized by history of a depressive disorder during the last 12 months

More than 40.0% (n = 3027) of participants without a recent history of DD were not on any medication, while this percent was 17.2% (n = 309) among patients with a recent history of DD. More than 51.7% of individuals with a recent history of DD and 76.6% of participants without a recent history of DD were on less than two different drugs at the time of the study (Fig. 1).

**Fig. 2:**
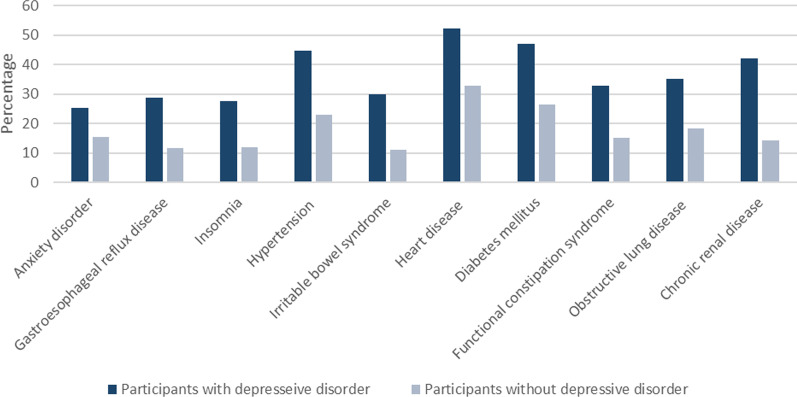
Polypharmacy prevalence among participants with or without a recent history of depressive disorders categorized with top ten most common underlying diseases in the PCS population (from left to right)

The top ten common diseases among the PCS population were anxiety disorder (29%), gastroesophageal reflux disease (25%), insomnia (19%), hypertension (16%), irritable bowel syndrome (11%), heart disease (10%), diabetes mellitus (9%), functional constipation syndrome (8%), obstructive lung disease (3%) and chronic renal disease (1%), respectively (with exclusion of having recent history of depressive disorder). Among participants with each of these underlying diseases, the prevalence of polypharmacy was statistically significantly higher among those with recent history of a depressive disorder than those who had not any depressive disorder within the last 12 months (all p-values were less than 0.05; Fig. 2).

According to multivariable logistic modeling, to identify determinants of higher prevalence of polypharmacy among patients with a recent history of DD, patients who were suffering from metabolically unhealthy overweight have a prevalence of 1.85 times of that in patients without unhealthy overweight (95% CI 1.25, 2.74). Surprisingly, not being a current cigarette smoker was a significant determinant for a higher prevalence of polypharmacy among patients with a history of depressive disorder during the last 12 months (OR: 1.77; 95% CI 1.02, 3.10; Table 2).

**Table 2:** Associated factors with the prevalence of polypharmacy among patients with a history of depressive disorder during the last 12 months

Factor	Crude odds ratio (95% CI)	Adjusted odds ratio (95% CI)
Being female	2.40 (1.81, 3.20)	1.51 (1.06, 2.14)
Being fars*	1.49 (1.18, 1.87)	1.52 (1.18, 1.97)
Being unhealthy overweight	2.10 (1.47, 3.01)	1.85 (1.25, 2.74)
Having low physical activity	2.11 (1.68, 2.63)	1.74 (1.36, 2.31)
Being in higher socioeconomic quartile	1.18 (0.91, 1.54)	1.40 (1.03, 1.86)
Having an additional comorbidity	0.79 (0.60, 1.04)	1.72 (1.60, 1.87)
Being cigarette non-smoker	3.09 (1.96, 4.87)	1.77 (1.02, 3.10)
Being tobacco smoker, ever	1.62 (1.29, 2.02)	1.24 (0.96, 1.60)

CI, confidence interval

*Reference is other ethnicities

Alimentary tract and metabolism drugs class (41.3%) was the most prevalent class after cardiovascular drugs (49.7%) among patients older than 59 years. Drugs related to the genitourinary system (55.4%) and cardiovascular drugs (37.4%) were the most prevalent drug classes used by women who had a recent history of DD, while nervous system drugs (29.1%) and cardiovascular drugs (27.8%) were the most common classes among males with a recent DD (Table 3).

**Table 3:** ATC classification (1st level) of drugs used by patients with a recent history of depressive disorders

Drug class*	Total n (%)	Men n (%; 95% CI)	Women n (%; 95% CI)	60 years or older n (%; 95% CI)
G	703 (39.1)	7 (1.3; 0.52, 2.68)	696 (55.4; 51.4, 59.7)	111 (22.3; 18.4, 26.9)
C	620 (34.4)	150 (27.8; 23.6, 32.7)	470 (37.4; 34.1, 41.0)	247 (49.7; 43.7, 56.3)
A	562 (31.2)	133 (24.7; 20.7, 29.2)	429 (34.2; 31.0, 37.6)	205 (41.3; 35.8, 47.3)
N	525 (29.1)	157 (29.1; 24.8, 35.1)	368 (29.3; 26.4, 32.5)	142 (28.6; 24.1, 33.7)
B	510 (28.3)	79 (14.7; 11.6, 18.3)	431 (34.3; 31.2, 37.7)	157 (31.6; 26.8, 36.9)
M	258 (14.3)	50 (9.3; 6.9, 12.2)	208 (16.6; 14.4, 19.0)	119 (23.9; 19.9, 28.7)
H	238 (13.2)	46 (8.5; 6.3, 11.4)	192 (15.3; 13.2, 17.0)	89 (17.9; 14.4, 22.0)
R	122 (6.8)	31 (5.7; 3.9, 8.2)	91 (7.3; 5.8, 8.9)	41 (8.3; 5.9, 11.2)
S	20 (1.1)	4 (0.7; 0.20, 1.9)	16 (1.3; 0.7, 2.1)	10 (2.0; 1.0, 3.7)
L	7 (0.4)	2 (0.4; 0.04, 1.3)	5 (0.4; 0.1, 0.9)	2 (0.4; 0.1, 1.5)

ATC: anatomical therapeutic chemical; A: alimentary tract and metabolism; B: blood, and blood-forming organs; C: cardiovascular system; G: genitourinary system; H: systemic hormonal preparations, excl.: Sex hormones and insulins; L: antineoplastic and immunomodulating agents; M: musculoskeletal system; N: nervous system; R: respiratory system; S: sensory organs

*Ranked based on the total prevalence

The non-selective monoamine reuptake inhibitors and selective serotonin reuptake inhibitors were used comparably in females (around 14.0%), males (around 41.0%), and elderly people (around 20.0%) The other antidepressants were used 9.2%, 36.6% and 16.1% accordingly. (Table 4).

**Table 4:** ATC classification of antidepressants used by patients with a recent history of depressive disorders

Drug class	Total n (%)	Men n (%; 95% CI)	Women n (%; 95% CI)	60 years and older n (%; 95% CI)
N06AA	402 (22.4)	224 (41.6; 36.3, 47.4)	178 (14.5; 12.5, 16.8)	98 (19.7; 16.0- 24.0)
N06AB	401 (22.3)	219 (40.6; 34.2, 46.4)	182 (14.2; 12.2, 16.4)	100 (20.1; 16.4; 24.5)
N06AX	312 (17.4)	197 (36.6; 31.6, 42.0)	115 (9.2; 7.6, 11.0)	80 (16.1; 12.8- 20.0)

ATC: Anatomical therapeutic chemical; N06AA: non-selective monoamine reuptake inhibitors; N06AB: selective serotonin reuptake inhibitors; N06AX: other antidepressants

**Fig. 3:**
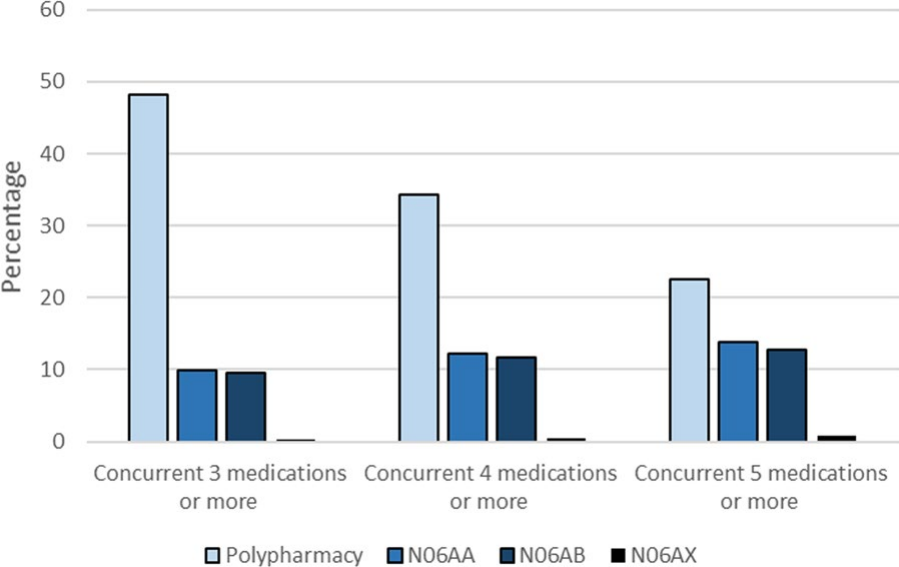
Percentage of ATC classes of antidepressants (2nd level) use among patients with a recent history of depressive disorders with different definitions of polypharmacy; concurrent use of 3 or more medications, 4 or more medications and 5 or more medications. N06AA: non-selective monoamine reuptake inhibitors; N06AB: selective serotonin reuptake inhibitors; N06AX: other antidepressants

A similar rate of non-selective monoamine reuptake inhibitors and selective serotonin reuptake inhibitors use was seen among participants with a recent history of depressive disorders who had concurrent use of 3 or medications and also among those with 4 or more medications and those with 5 or more medications (the cutoff used in this report for polypharmacy). Percentage of application of each of these two drug classes was about 10 percent among those with concurrent use of 3 or more medications, 12 percent among participants with concurrent use of 4 or more medications and 13 percent among participants with concurrent use of 5 or more medications (Fig. 3).

## Discussion

This population-based study aimed to investigate the prevalence and determinants of polypharmacy in a less developed region in southwestern Iran. The prevalence of depressive disorders during the last 12 months was estimated at 19.4%. Polypharmacy among patients with a recent history of DD was about 22%, which is two times higher than the prevalence in participants without such history. This study revealed that gender, ethnicity, physical activity, being overweight, SES, cigarette and tobacco smoking, and the presence of multimorbidity were independently associated with polypharmacy. After excluding the cardiovascular system drugs class, drugs related to the nervous system, the genitourinary system, and the alimentary tract and metabolism were the most common drug classes used by men, women, and elderly people, respectively. Consumption of both non-selective monoamine reuptake inhibitors and selective serotonin reuptake inhibitors among men was around three times more prevalent than that in women.

Although studying polypharmacy needs to separate its necessary type from the inappropriate forms, we know that polypharmacy, in a considerable share, is an indication of inappropriate or avoidable medication use, or some possible malpractices in the drug prescription [1, 21, 22]. Moreover, it may be partly a result of the unavailability of the combined drugs recently designed and approved [23]. On the other hand, regardless of its appropriateness, polypharmacy is a leading cause of non-adherence to the medication therapy [24]. Non-adherence to antidepressants is one of the challenging issues in the management of DDs, as it may complicate the psychopharmacotherapy and lead to a higher chance of relapse [12].

In line with the previous evidence, in this study the prevalence of polypharmacy among patients with DD was higher than that in participants without a recent history of DD [9]. Some authors have argued that this higher prevalence is fully explained by the higher risk of comorbidities among patients with depression [11], but others have shown that even after adjustment for comorbidities the chance of polypharmacy in patients with DD is higher than that in patients without DD [25]. In this study, the prevalence of polypharmacy among patients with both multimorbidity and history of DD was about 23% compared to about 14% among multimorbid patients without a history of DD. It may be a result of the health-seeking and medication utilization behaviors of patients that their DD has been diagnosed in a society without any active specialized psychiatric clinic. We also showed that after adjustment for age and gender, having a recent history of DD increases the prevalence of polypharmacy by more than 22 times. Accordingly, psychiatrists should be aware of this huge higher chance of polypharmacy among younger females with a recent history of DD and consider combination or more simple medication regimes, as well as deprescribing and other approaches to reduce and prevent polypharmacy among them [21].

This study revealed that the prevalence of polypharmacy is higher in women. A nationwide study from Sweden has shown that psychiatric polypharmacy among women was higher than men [26]. Other studies have shown that the prevalence of polypharmacy among women, regardless of their status regarding depression, is higher than men [25]. It may be a result of their healthcare-seeking behaviors such as their higher rates of adherence to the medication therapy and self-medication [27]. It may also be a result of higher rates of multiple episodes or persistent DD among women compared to men.

Patients with Fars ethnicity have a higher prevalence of polypharmacy compared with others. Previous studies have shown that minorities and deprived subpopulations experienced higher rates of polypharmacy. It may be a result of their less access to and utilization of healthcare services such as pharmacy, and their less adherence to medication therapy.

Patients with the highest SES have a higher prevalence of polypharmacy. Although healthcare services are provided by the public sector in the study setting as specialized services are not available in this setting, higher SES may result in higher utilization of specialized services available in neighboring cities while in other SES a higher chance of untreated DD is probable. They may have a higher rate of adherence to the medication therapy or may use more OTC drugs such as vitamins, and also, they may be more affected by the direct-to-consumer (DTC) marketing because of the affordability of drugs not covered by insurance.

According to the study findings, low physical activity, unhealthy overweight, and tobacco smoking increase the prevalence of polypharmacy among patients with a history of DD. Such patients may have more inappropriate health-related behaviors and a probable lower adherence to prescribed therapies. Besides, these factors are risk factors for several chronic diseases. These chronic diseases their selves could lead to increased risk of development of depressive disorder or even deterioration of the severity of the already existing depressive disorder and therefore higher rate of polypharmacy[28, 29]. Although we adjusted for multimorbidity, as the number of comorbidities, we were unable to adjust for the severity of patients’ comorbidities. Therefore, this finding may be a residual confounding effect of the patients’ comorbidities. In the case of unhealthy overweight, it may be evidence of an undiagnosed symptomatic pathological mechanism that results in more medication use.

We showed that more than 55% of women with a history of DD were using the genitourinary system drugs class compared with less than 2% in men. A study from Colombia has reported that more than 50% of women older than 40 years were suffering from the genitourinary syndrome of menopause [30]. Sundbom et. al. [26] have shown that among women with DD, drugs related to the genitourinary system were the 3rd common prescribed drug class. Psychiatrists may also need to consider asking about genitourinary diseases from female patients and adopt the therapeutic approaches that they chose. It may be improving the patients’ medication adherence and consequently decrease the rate of relapse.

Among men, the prevalence of use of almost all of the drug classes was 1.5 to 4 times lower than women, except for the nervous system drug class. Lower drug utilization among men is consistent with previous reports from other regions. However, it may be evidence of medication underuse among men. In case of a similar rate of utilization of the nervous system drugs class, considering a significantly lower rate of medication underuse among men compared with women, it may be a piece of evidence that in this setting men who are suffering from DD may also be suffering from other nervous system diseases such as epilepsy, neurodegenerative diseases, etc. that are more common in males [31]. Further studies to investigate this issue are recommended.

Surprisingly, the prevalence of using antidepressants was higher among men compared with women. It may be a result of differential approaches preferred by the psychiatrists or the patients themselves to manage the DD in males and females [27]. It may also be a result of a higher share of milder disorders in females [24].

The study findings showed that the prevalence of prescribing/utilization of both non-selective monoamine reuptake inhibitors (or Tricyclic antidepressants (TCAs)), and selective serotonin reuptake inhibitors (SSRI) were similar. A study from England reported a completely different pattern as more than 85% of patients were only on SSRI [24]. This finding may be an indication of malpractice, a probable high rate of severe DD, or a different pattern of the effectiveness of available SSRIs in our setting, as SSRIs are typically used as the first-line psychopharmacotherapy to manage DD [32]. A similar result has been reported from Germany [14]. However, such a high rate of use of TCAs leads to a higher rate of drug side effects and consequently lower adherence to medication therapy or a higher rate of polypharmacy [15]. Further studies on the patterns of prescription and utilization of antidepressants are needed.

This study used population-based but cross-sectional data. We were not able to determine the temporality of the polypharmacy and time-dependent variables, such as unhealthy overweight, that we identified as independent determinants of polypharmacy. Also, we were unable to investigate the effects of untreated or relapsed depression on polypharmacy. Another limitation of the study was our age-restricted sample excluding populations younger than 40 years old. Furthermore, this cross-sectional nature of the study limited us for the cause-effect analyses and we were not able to determine whether this is the depressive disorder that led to higher prevalence of polypharmacy among those with a medical disorder for example diabetes mellitus or conversely, it is the diabetes that is responsible for use of more medications among participants with recent depressive disorder. Besides, a high number of diseases has been included in the Pars Cohort Study database and there were small number of patients with some of these underlying diseases; accordingly, this issue limited us from including having different diseases in the multivariate analysis and the interaction between having recent depressive disorder and other diseases was not assessed in our main modelling. However, we designed Fig. 2 to depict an overview of the association of depression and polypharmacy prevalence among patients with different medical diseases.

## Conclusion

The prevalence of polypharmacy is high among patients with a recent history of depressive disorder. Female patients of higher SES class with less physical activity, and who are tobacco users are at higher risk of being polymedicated. General physicians and psychiatrists should be aware of this higher risk of polypharmacy among such patients and should be trained to consider appropriate approaches to reduce and prevent polypharmacy among such patients. The pattern of antidepressant use should be investigated in future studies in Iran.

## Data Availability

Data is available upon request by the PCS board (please see https://persiancohort.com/).
